# ATR-IR Spectroscopy Application to Diagnostic Screening of Advanced Endometriosis

**DOI:** 10.1155/2022/4777434

**Published:** 2022-06-06

**Authors:** Izabela Kokot, Sylwester Mazurek, Agnieszka Piwowar, Roman Szostak, Marcin Jędryka, Ewa Maria Kratz

**Affiliations:** ^1^Department of Laboratory Diagnostics, Division of Laboratory Diagnostics, Faculty of Pharmacy, Wroclaw Medical University, Borowska Street 211A, 50-556 Wroclaw, Poland; ^2^Faculty of Chemistry, University of Wrocław, F. Joliot-Curie 14, 50-383 Wrocław, Poland; ^3^Department of Toxicology, Faculty of Pharmacy, Wroclaw Medical University, Borowska Street 211, 50-556 Wroclaw, Poland; ^4^Department of Oncology, Gynecological Oncology Clinic, Faculty of Medicine, Wroclaw Medical University, Hirszfeld Square 12, 53-413 Wroclaw, Poland; ^5^Department of Oncological Gynecology, Wroclaw Comprehensive Cancer Center, Hirszfeld Square 12, 53-413 Wroclaw, Poland

## Abstract

Endometriosis is one of the most common gynecological diseases among young women of reproductive age. Thus far, it has not been possible to define a parameter that is sensitive and specific enough to be a recognized biomarker for diagnosing this disease. Nonspecific symptoms of endometriosis and delayed diagnosis are impulses for researching noninvasive methods of differentiating endometriosis from other gynecological disorders. We compared three groups of individuals in our research: women with endometriosis (E), patients suffering from other gynecological disorders (nonendometriosis, NE), and healthy women from the control group (C). Partial least squares discriminant analysis (PLS-DA) models were developed based on selected serum biochemical parameters, specific regions of the serum's infrared attenuated total reflectance (FTIR ATR) spectra, and combined data. Incorporating the spectral data into the models significantly improved differentiation among the three groups, with an overall accuracy of 87.5%, 97.3%, and 98.5%, respectively. This study shows that infrared spectroscopy and discriminant analysis can be used to differentiate serum samples among women with advanced endometriosis, women without this disease, i.e., healthy women, and, most importantly, also women with other benign gynecological disorders.

## 1. Introduction

Endometriosis (E) is a benign gynecological disease in which endometrial tissue grows outside the uterus and acts as a eutopic endometrium, causing local inflammation and fibrosis. The consequences of this process include chronic pain and changes leading to infertility [1]. Despite numerous studies, no clear cause for the development of endometriosis has yet been identified, suggesting multifactorial pathogenesis [2, 3]. This is the fundamental problem that makes proper disease diagnosis and treatment difficult since no highly specific diagnostic marker exists. So far, laparoscopy has been recognized as the golden standard of endometriosis diagnostics. However, the current ESHRE guideline (2022) does not support this recommendation anymore, and now, laparoscopy is recommended only for patients with negative imaging results and/or where empirical treatment was unsuccessful or inappropriate. However, members of the Endometriosis Guideline Core Group emphasize that there is still an urgent need for more research to gain more clarity on the most appropriate diagnostics, including laboratory diagnostics [4]. Therefore, there is a great need for a broadly understood, noninvasive diagnosis of endometriosis.

Infrared (IR) and Raman spectroscopy can provide information enabling correct and detailed characteristics of different diseases [5]. Changes in tissues, cells, and body fluids due to lesions and infection are reflected in the spectroscopic data of biological material. Thus, disease-specific spectral biomarkers of blood or serum could support effective medical diagnostics and have a significant impact on rapid screening for potential patients in large-population tests [6–8].

Attenuated total reflection (ATR) allows IR data of samples to be collected in their native state. The major advantage of this method is that samples can be examined directly in the solid or liquid state without further preparation. For blood serum samples, spectra can be recorded directly for hydrated samples, as they are, or for thin films obtained by drying the fluid on an ATR crystal. In the former case, strong water bands obscure the shape of signals from the remaining components present in the sample. Formally, water contributions can be subtracted from serum spectra, but some specific features in a difference spectrum can be skewed due to hydrogen bonding stabilizing the molecules' structures. The most common procedure applied in blood spectroscopy is collecting IR data from dried, thin films. However, drying serum samples on the ATR crystal is a time-consuming procedure. As an alternative, the ATR spectra of the freeze-dried or lyophilized sera can be utilized. The quality of classification based on IR data depends on the sample storage, drying processes, and other preanalytical factors [9].

Spectroscopic methods usually generate large datasets consisting of thousands of variables, absorbances in the case of ATR spectroscopy. Very often, a strong correlation is observed between absorbances registered at different wavenumbers. Additionally, spectra can be distorted by spectral noise, depending on the instrumental factors and sample characteristics. Therefore, analysis is often supported by multivariate methods to extract relevant information from spectral data, reduce their dimensionality, and avoid overfitting. By applying principal component analysis (PCA) or discriminant analysis (DA), it is possible to distinguish among samples belonging to particular groups [10]. A conjunction of ATR spectroscopy and multivariate modeling techniques has found applications in molecular fingerprinting of disease development, including of breast, brain, and ovarian cancers [11–16]. Classification has been reported of sera samples from patients with Salmonella [17] or viral infections [18–20]. Serum spectra were also used to determine levels of total protein [21], glycated albumin [22], lipidic parameters [23, 24], and glucose [25].

In this study, we present multivariate models that can discriminate among advanced endometriosis, nonendometriosis, and healthy controls by applying previously determined biochemical parameters of serum samples [2, 26] and their ATR spectra. In a commonly used approaches, two groups, i.e., patients and healthy controls, are usually taken into account [27, 28]. However, in our research, two groups of women suffering from benign pathologic conditions, one with advanced endometriosis and one for which endometriosis was excluded, were compared with a group of healthy women without any symptoms of inflammation or medical history of endometriosis, to select biomarkers allowing for discrimination among these three groups of women.

## 2. Materials and Methods

### 2.1. Serum Samples

Serum samples from patients with advanced endometriosis (E, *n* = 29, interquartile range of age 31.0–43.0) and without endometriosis (reference group; nonendometriosis, NE, *n* = 24, interquartile range of age 33.0–43.5) were collected at the Department of Oncological Gynecology, Wroclaw Comprehensive Cancer Center. The control group comprised healthy female volunteers (control group, C, *n* = 18, interquartile range of age 35.0–41.0). The E and NE groups had undergone surgical interventions, mainly laparoscopy; following histological verification, they were assigned to the proper group. Patients belonging to E group had advanced endometriosis, corresponding to the revised American Fertility Society classifications of stages III (*n* = 12) and IV (*n* = 17). Women in the NE group were histologically confirmed to have leiomyomas, benign ovarian cysts or severe cervical dysplasia, and cervical intraepithelial neoplasia grade 3 (CIN 3). The control group consisted of healthy, nonpregnant women of reproductive age who were premenopausal, lacked gynecological problems, had no history or symptoms related to endometriosis, and had no symptoms of inflammation, therefore, they were not qualified for laparoscopy. The main exclusion criterion for all groups was cancer, present, treated, or past. Another exclusion criteria were menopause and previous hysterectomy. Due to the homogeneity of the study group (E), the exclusion criterion was endometriosis in the cyst of the abdominal integuments and the postoperative scar and adenomyosis. Only patients with confirmed stage III or IV of endometriosis according to the rAFS classification were included in the study. The reference group (NE) included only patients with histopathologically excluded endometriosis, but with mild gynecological disorders. Women from the control group were recruited from among employees of the Wroclaw Medical University and from our friends. All of the participants were of a similar age and had comparable body mass indexes.

All information regarding blood collection and handling was described in our previous work [2, 26]. The present study was conducted in agreement with the Helsinki II Declaration, and the protocol was approved by the Bioethics Human Research Committee of Wroclaw Medical University (No. 231/2019, No. 634/2019, and No. 685/2019). All of the subjects gave written and informed consent prior to their participation in the study. All of the biochemical analyses were carried out in accordance with the manufacturers' instructions.

### 2.2. Biochemical Analysis

High sensitive interleukin 1*β* (hsIL-1*β*), interleukin 6 (IL-6), chitinase-3-like protein 1 (YKL-40), sirtuins (SIRTs: SIRT3, SIRT5, and SIRT6), and telomerase (TE) levels were determined with commercially available ELISA tests. Human IL-1*β* ELISAPRO kits (MABTECH AB, Nacka Strand, Stockholm, Sweden) were used to measure hsIL-1*β* concentrations. High Sensitivity ELISA kit (The Covalab, Villeurbanne, France) and Human Chitinase-3-like Protein 1 ELISA Kit (Bioassay Technology Laboratory, Shanghai, China) were used for determining IL-6 and YKL-40 concentrations, respectively. Sirtuin concentrations were measured with Human Sirtuin 3 ELISA Kit, Human Sirtuin 5 ELISA Kit, and Human Sirtuin 6 ELISA Kit (Bioassay Technology Laboratory, Shanghai, China), and TE levels were determined using Human Telomerase ELISA Kit (CUSABIO Technology LLC, Wuhan, China). A Mindray-96A ELISA plate reader (Mindray, Shenzhen, China) was used to measure the concentrations of these inflammatory parameters. C-reactive protein (CRP) and immunoglobulin G (IgG) concentrations were measured using the immunoturbidimetric method, highly sensitive for CRP (U-hs DiaSys Diagnostic Systems GmbH, Holzheim, Germany) and immunoglobulin G (FS DiaSys Diagnostic Systems GmbH, Holzheim, Germany), respectively, using the biochemical analyzer Konelab 20i® (ThermoScientific, Vantaa, Finland). This analyzer was also used to determine the levels of total antioxidant status (Randox TAS Kit, Crumlin, United Kingdom), glucose (GLU), total protein (T-P), albumin (ALB), total bilirubin (T-BIL), uric acid (UA), iron (Fe) (Thermo Scientific, Vantaa, Finland), calcium (Ca), magnesium (Mg), total cholesterol (T-CHOL), triglycerides (TG), and high-density lipoprotein (HDL) cholesterol (DiaSys Diagnostic Systems GmbH, Holzheim, Germany). LDL (low-density lipoprotein) cholesterol was calculated using Friedewald's formula. Carcinoma antigen 125 (CA 125), prolactin (PRL), and estradiol (E2) concentrations were measured by Cobas® 6000 analyzer (Roche, Mannheim, Germany). The concentrations of advanced protein oxidation products (AOPP) were measured according to the method of Witko-Sarsat et al. [29], and the ferric-reducing antioxidant power (FRAP) was measured using Benzie and Strain's method [30]. Levels of these parameters were measured using the UV/Vis spectrophotometer (UV-6300PC, VWR, Shanghai, China).

### 2.3. Spectroscopic Conditions

Attenuated total reflection (ATR) FTIR spectra of serum samples were recorded with an iS50 FTIR spectrometer (Thermo Nicolet, Madison, WI, USA) using a single-reflection Golden Gate (Specac, Slough, UK) diamond accessory. Measurements were performed using a KBr beamsplitter and a DTGS detector. Interferograms were averaged over 128 scans. Next, they underwent Happ-Genzel apodization and Fourier transformation using a zero-filling factor of 2 to give spectra in the 400-4000 cm^−1^ range with a resolution of 4 cm^−1^. A single FTIR spectrum of serum consisted of 7,469 absorbance points.

Before measurement, the frozen serum samples were thawed at room temperature for 30 min. An aliquot of 10 *μ*L of serum was deposited on ATR crystal and nitrogen-dried over 60 min to obtain a thin film of biological material to analyze. After each measurement, the crystal was cleaned with methanol. Serum samples belonging to various groups of women were measured alternately.

### 2.4. Computational Analysis

The studied datasets, i.e., matrices of the biochemical diagnostic parameters (71 × 29) and the absorbance intensities of the ATR spectra (71 × 7469), were analyzed by applying PCA and discriminant analysis using partial least squares regression through PLS-Toolbox in MATLAB (ver. R2010a, MathWorks, Natwick, MA, USA). The second derivatives of spectral data were computed utilizing the Savitzky–Golay algorithm, applying third-degree polynomial and 15-point windows. Biochemical data were autoscaled before chemometric modeling, while the ATR spectra were mean-centered. The constructed models were cross-validated by applying the leave-one-out procedure. General least squares weighting (GLSW) was applied to PCA performed on the serum parameters [31]. Variables were selected by applying the interval PLS (iPLS) algorithm, as implemented using PLS-Toolbox, working in a forward mode. The mean spectra, together with the standard deviation (SD) of absorbance at each wavenumber, were computed to determine and compare the average IR spectra for all three sample groups.

### 2.5. Principal Component Analysis (PCA)

Data originating from modern spectrometers are characterized by highly redundant information. In typical analyses, the number of obtained variables, e.g., absorbance at a given wavenumber, is much greater than the number of analyzed objects. PCA transforms correlated explanatory variables into new ones that do not show any correlation. These new variables, i.e., principal components (PCs), are linear combinations of explanatory variables, and each PC is orthogonal to the others. The successive PCs explain decreasing variance present in the data not accounted for by previous PCs. When a specific variability resulting from the nature of the investigated objects is greater than undesirable random variability, only the *k*-first PCs are considered [32]. Therefore, using PCs can reduce data dimensionality significantly without information loss.

PCA decomposes the *X* data matrix, containing *n* rows (objects) and *m* columns (variables), into two smaller matrices:

(1) $$\begin{array}{c} X_{n,m}=T_{n,k}\times {P^{T}}_{k,m}+E_{n,m}, \end{array}$$

where *T* (the scores matrix) describes the relations among the samples, *P* (loadings) provides the mutual dependencies between variables, and *E* shows differences between the data matrix values and those obtained from the product of matrices *T* and *P*.

### 2.6. Partial Least Squares Discriminant Analysis

Partial least squares discriminant analysis (PLS-DA) is a chemometric technique for separating groups of samples by combining a dataset matrix (*X*) with class membership (*Y*). This approach is aimed at maximizing the covariance between the independent variables *X* and the corresponding dependent variable *Y* of highly multidimensional data by finding a linear subspace of the explanatory variables. This new subspace allows *Y* to be predicted based on a reduced number of PLS factors or latent variables (LV). These factors describe the behavior of dependent variables and include a subspace onto which independent variables are projected [33, 34]. The main advantage of PLS-DA is its ability to handle highly collinear and noisy data, which are very common outputs from spectroscopic measurements or metabolomics experiments. This technique provides a visual interpretation of complex datasets through low-dimensional, easily interpretable score plots that illustrate the separation between different groups [33].

### 2.7. Classifier Evaluation Criteria

Different criteria can be used to evaluate the quality of classifiers. In our analysis, classification accuracy, sensitivity, specificity, and receiver operating characteristic curves (ROC) were used to characterize the performance of the obtained PLS-DA models. In medical applications, the model characterized by higher area under the ROC is better suited for distinguishing patients from healthy subjects. “Positive” and “negative” results are classification predictions obtained from the model. “True” and “false” are the actual data. The sensitivity, specificity, and accuracy were calculated using the following equations:

(2) $$\begin{array}{c} \text{Sensitivity}=\frac{\text{TP}}{\text{TP}+\text{FN}}, \end{array}$$

(3) $$\begin{array}{c} \text{Specificity}=\frac{\text{TN}}{\text{TN}+\text{FP}}, \end{array}$$

(4) $$\begin{array}{c} \text{Accuracy}=\frac{\text{TP}+\text{TN}}{\text{TP}+\text{TN}+\text{FP}+\text{FN}}, \end{array}$$

where TP and TN denote the true-positive and true-negative values and FP and FN represent false-positive and false-negative values, respectively.

## 3. Results and Discussion

Diagnosing endometriosis based on parameters of peripheral blood serum is not straightforward. The disease's development appears directly related to inflammatory processes, and since there is no specific biomarker [35], only a combination of commonly determined blood biochemical markers may allow endometriosis to be distinguished from other inflammatory conditions and, in the future, increase the chances of detecting endometriosis in large-scale tests of serum samples from women.  Table 1 provides values of selected biochemical parameters for the three groups of subjects, and Table S1 in the Supplementary Materials contains a complete list of the examined serum parameters.

### 3.1. Multivariate Analysis of Serum Parameters

Our recent studies discussed the importance of selected blood serum parameters for advanced endometriosis diagnostics [2, 26]. The most promising serum parameters as markers of inflammation and oxidative-antioxidant balance were interleukin 6, prolactin, CA 125, FRAP, telomerase, and advanced protein oxidation products. Although these parameters are not specific to advanced endometriosis, they can serve as useful noninvasive diagnostic tools for identifying patients with high risk of developing advanced endometriosis. This itself is a challenge.

Principal component analysis (PCA) was performed on an autoscaled matrix of parameters without any additional pretreatment. The distribution of the objects in the PC1/PC2 coordination system, as presented in Fig. S1 in the Supplementary Materials, showed no specific grouping of samples, and combination of higher PCs did not improve the separation. Separation of the three samples groups became clearer when applying general least squares weighting (GLSW) which resulted in the expected sample arrangement in the PCA score plot. This plot together with the loadings on PCs is presented in Fig. S2 in the Supplementary Materials. Given these plots, the parameters displaying the most pronounced impact on differentiation among serum samples were CA 125, immunoglobulin G, albumin, magnesium, hsIL-1*β*, and FRAP. Our findings seem to be particularly important considering that during the development of inflammation, an increase in the serum concentration of inflammatory markers CA 125 and IgG and the proinflammatory cytokine hsIL-1*β* is observed, with a simultaneous decrease in the level of albumin, i.e., acute phase protein. Increased FRAP, as one of the antioxidant markers reflecting blood plasma's antioxidant capacity, is associated with elevated free radical production; their concentration increases significantly in inflammatory conditions. On the other hand, magnesium deficiency may also be associated with inflammation and increased concentration of free radicals. Inflammatory mediators and free radicals could induce oxidative DNA damage [36, 37]. Previous studies have suggested that persons with endometriosis experience vascular inflammation [38, 39]. Magnesium relaxes smooth muscle [40, 41] and thus may be related to endometriosis through its influence on retrograde menstruation [41]. The obtained PCA score plots are even more important because PCA belongs to a group of an unsupervised methods and the algorithm does not take class affiliation into account during matrix decomposition.

**Figure 1:**
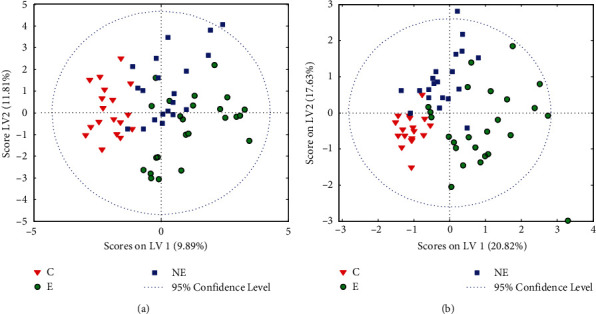
Score plots for PLS-DA modeling of biochemical data from serum samples: (a) all parameters included (*n* = 29) and (b) iPLS variable selection (*n* = 6). E: endometriosis; NE: nonendometriosis; C: control group of healthy women.

In the next step, the PLS-DA model was constructed by applying the same dataset of serum parameters. The model including all available parameters separated the samples belonging to E, NE, and controls relatively well. The PLS-DA scores are shown in Figure 1, and Fig. S3 in the Supplementary Materials presents the variable importance in projection (VIP) scores. The latter plot indicates that CA 125, together with albumin and magnesium content, had the strongest impact on differentiation among the three studied groups. Our findings are in line with those of the previous studies, in which advanced endometriosis was associated with high serum CA 125 levels [36]. Due to the lack of a specific marker for endometriosis, CA 125 concentration in serum is considered an important prognostic factor in patients with endometriosis in clinical practice and should be considered when surgical treatment is suspected, particularly when assessing the disease's severity, the size of the lesion, and adhesions [42]. Interestingly, in comparison to PCA modeling, no pronounced differences were observed in sample classification when the GLSW pretreatment was used.

The interval PLS (iPLS) algorithm was applied to select variables, in order to reduce the dimensionality of the parameters' matrix and find the most significant determinants of serum. On this basis, six of the 29 diagnostic parameters were chosen. The obtained set, namely, CA 125, IgG, CRP, albumin, magnesium, and chitinase-3-like protein 1 (YKL-40), is quite similar to that one found using VIP scores. All of the selected parameters reflect the ongoing inflammation. It is believed that YKL-40 is a marker which excludes endometriosis, rather than confirms its presence or progression [2, 43]. The classifier constructed for four LVs (latent variables) applying selected inputs was characterized by accuracy of 91-92% (86-89%), sensitivity of 81-94% (75-94%), and specificity of 89-100% (87-95%), with the models' cross-validation results shown in parentheses. In our opinion, the results presented above clearly indicate the high clinical usefulness of the selected parameters for identifying advanced endometriosis diagnoses. Detailed characteristics of the obtained PLS-DA model are presented in Table 2 and Table S2 in the Supplementary Materials. The PLS-DA model's scores are shown in Figure 1, while plots of the receiver operating characteristic (ROC) curves, expressing the classification performance, are shown in Fig. S4 in the Supplementary Materials.

### 3.2. ATR Spectra of Serum

**Figure 2:**
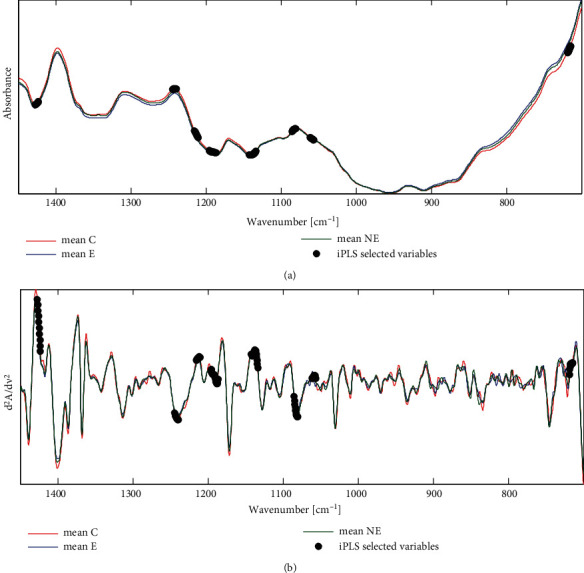
Average FTIR ATR spectra of sera in the 700-1450 cm^−1^ range for the three studied groups (a) and the second derivatives of the spectra (b); the black dots indicate variables selected by iPLS for the PLS-DA model. E: endometriosis; NE: nonendometriosis; C: control group of healthy women; cm^−1^: unit of the wavenumbers presented as the reciprocal centimeters.

In parallel with biochemical analysis, FTIR ATR spectra of 71 human sera were collected.  Figure 2 and Fig. S5 in the Supplementary Materials show the IR spectra of the samples from the three groups of women. Tentative assignments of the vibrational bands present in ATR spectra can be found elsewhere [44, 45]. The spectra of the E, NE, and C groups are very similar, and subtraction plots (Fig. S6 in the Supplementary Materials) indicate that their differences exceeded the standard deviation of absorbance intensity for the mean spectra only at particular wavenumbers.

Preparations of thin serum film onto an ATR crystal are not always reproducible and can result in nonequal sample deposition on the crystal, despite attempts to follow all established procedures. This can result in uneven drying of the samples, which generates undesirable spectral variation [46]. An effect of absorbance changes to IR spectra in a drying function is shown in Fig. S7 in the Supplementary Materials. The greatest variability of signal in the serum spectra was observed in the amide band regions. Drying resulted in strong changes to the intensity of the *ν*(OH) band in the 3000-3500 cm^−1^ range and the narrowing of the amide A band. Drying enhances spectral features originating from chemical components of serum, whereas strong water absorbance was observed in wet samples. These changes are observable for the amide I and II bands in the IR serum spectra, with maxima at about 1640 and 1540 cm^−1^, respectively. As is visible in Fig. S7 in the Supplementary Materials, changes to the water content influence the band positions, which, in the case of unequal drying of serum samples, can be a source of additional variability influencing the classification results. This effect also may be important when analyzing signal intensity. Taking our observations into account, and based on the experiences of other researchers, we standardized the conditions of the analysis process to obtain reliable and repeatable results. In a series of preliminary experiments, the biological samples were dried for 60 min. After this time, changes in the IR spectra's absorbance were much smaller than those observed after shorter time intervals were. The scores and loadings of the PCA obtained for the dried samples are plotted in Fig. S8 in the Supplementary Materials.

### 3.3. Multivariate Modeling of Spectral Data

Special attention was paid to the 700-1450 cm^−1^ range of the ATR spectra. As other studies pointed out, spectral ranges outside of amide band regions are better suited to discriminating between ill and healthy subjects [28, 47]. The second derivatives of ATR spectra without additional pretreatment were used to construct chemometric models. The score plots of PCA for this spectral region did not allow the three groups of serum samples to be separated when considering the first two principal components. Only adding the third and fourth PCs allowed healthy controls to be distinguished from the E and NE groups but without clear distinction between advanced endometriosis and nonendometriosis patients. The score plots of PCA are shown in Fig. S9 in the Supplementary Materials. Discriminant analysis resulted in a quite similar distribution of samples. The score plots obtained from PLS-DA (Fig. S10 in the Supplementary Materials) show a clear separation between control and ill patients; however, similarly to PCA, E and NE objects were mixed. This suggests that the region of sera spectrum applied for modeling contains characteristic features that are correlated with overall inflammatory conditions but does not enable the recognition of different inflammation sources.

The VIP score plots were used to improve the quality of discrimination among the three studied groups based on the serum's ATR spectra (Fig. S11 in the Supplementary Materials). This method can indicate variables with differing spectra among the three examined groups. However, models constructed based on manually selected inputs did not enable satisfactory separation between the E and NE groups. Cleaner separation between samples was obtained with the PLS-DA model built with variables selected by the iPLS procedure. Taking into account the dimensionality of the absorbance data matrix, 10-variable intervals, corresponding to the spectral resolution of the ATR spectra, were established during the procedure. Eight intervals within the analyzed spectral range, i.e., 80 points of spectral data, were selected as inputs, as highlighted in Figure 2. The variables selected by the iPLS cover spectral regions containing some characteristic vibrations in the IR spectra of serum. These include the peaks at 1056 and 1080 cm^−1^, characteristic of nucleic acids, the *ν*_as_(PO_2_^−^) vibrations of phospholipids or *ν*(C-O) of ribose, and the band at 1186 cm^−1^, which can be assigned to the C-O-C asymmetric vibrations of phospholipids, triglycerides, and cholesterol esters. The selected variables also included a peak at about 1137 cm^−1^, which can be assigned to the *ν*_as_(CO-O-C) vibration of glycan DNA and RNA and the *ν*(C-O) of ribose; the band at 1213 cm^−1^, characteristic of A-DNA, *ν*_as_(PO_2_^−^) and RNA vibrations; and a contribution at 1241 cm^−1^ from nucleic acids, the *ν*_as_(PO_2_^−^) vibrations and immunoglobulins [44, 45].

**Figure 3:**
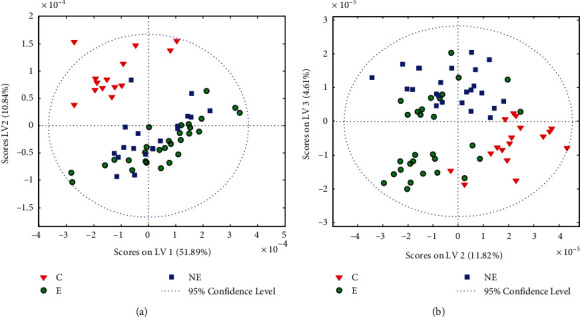
PLS-DA score plots obtained for the ATR spectra of the serum samples for the model applying the 700-1450 cm^−1^ range (a) and the model with variables selected via iPLS (b). E: endometriosis; NE: nonendometriosis; C: control group of healthy women.

In the resulting model, the two first latent variables described about 80% of the total variance present in spectral data, versus 63% by a model built without variable selection. The PLS-DA scores are presented in Figure 3; the ROC plots obtained for the developed model are shown in Fig. S12 in the Supplementary Materials. The modeling parameters are gathered in Table 2 and S2 in the Supplementary Materials. The model constructed using five PLS factors was characterized by accuracy of 97-99% (93-94%), sensitivity of 96-100% (88-92%), and specificity of 98-100% (93-96%) for the three studied groups; the cross-validation results are shown in parentheses. The quality parameters determined for the constructed classifier were significantly higher than those obtained from modeling biochemical data, and the overall accuracy reached 97% (Table 2).

The obtained results show compatibility between the biochemical and spectral data for the three studied patient groups, indicating that changes to the chemical composition of serum samples due to inflammatory conditions in advanced endometriosis and nonendometriosis patients have straightforward effects on their ATR spectra. Even when such differences are quite subtle, the variability present in spectral data can be separated effectively by PLS-DA, making vibrational spectroscopy a potential tool for detecting advanced endometriosis.

### 3.4. Models Based on Fused Data

**Figure 4:**
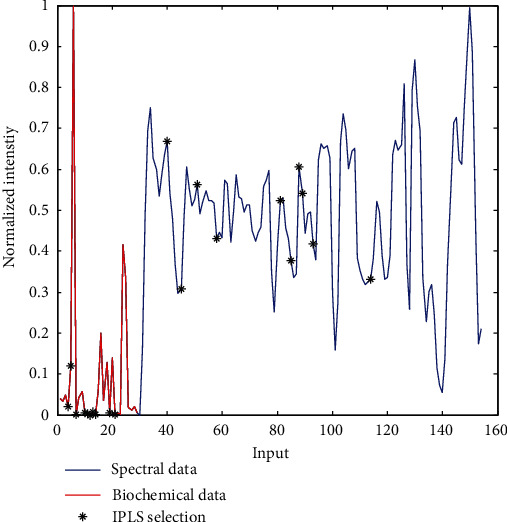
Representative input for fused data.

It seems justified to check whether classification models built using both biochemical and spectral data from serum samples would allow for better separation of the analyzed patient groups. This is not a straightforward operation because biochemical and spectral data differ. First, the number of biochemical parameters is orders of magnitude smaller than the number of points in the analyzed spectra. Second, their values also differ by orders of magnitude. Third, a noticeable proportion of spectral data provides no useful information due to spectral noise. To select a set of IR intensities representing the 700-1450 cm^−1^ range of ATR spectra, PCA was performed on a transposed matrix of the spectra's second derivatives. The original data were reduced by a factor of 10 after selecting evenly distributed points from each quadrant of the PC1/PC2 scores plot (Fig. S13 in the Supplementary Materials). Next, range scaling was applied to obtain a fused dataset containing biochemical parameters values normalized between 0 and 1 as well as intensities at selected wavenumbers. A representative input is presented in Figure 4.

Prior to modeling, the iPLS algorithm was used again to select the most relevant variables in the matrix of fused data. Absorbances at 10 wavenumbers (731, 747, 796, 834 1060, 1078, 1094, 1100, 1125, and 1243 cm^−1^) were selected by iPLS from the spectral portion of the combined dataset (Figure 4). Among them, the contributions can be distinguished from the C-C and C-O vibrations of carbohydrates (1094 and 1100 cm^−1^), features originating from nucleic acids and phospholipids (intensities at 1060 and 1078 cm^−1^) and immunoglobulins (band at 1243 cm^−1^) [44, 45]. Interestingly, among the 10 selected biochemical parameters of serum, three, CA 125, albumin, and magnesium, were the same as those selected for the classifier constructed for biochemical data, which seems to confirm their crucial role in separating the three patient groups. The remaining selected parameters include prolactin, total antioxidant status (TAS), total protein (T-P), total bilirubin (T-BIL), calcium (Ca), uric acid (UA), and hsIL-1*β*. These parameters are related to both the oxidative-antioxidant balance and inflammation state. Particular attention, except CA 125, should be paid to prolactin, which has a pleiotropic effect on the human body. Its most important functions are related to reproduction, calcium metabolism, osmoregulation, and behavior. Prolactin has an immunostimulatory effect, including promoting autoimmunity, although it cannot initiate an immune reaction itself; rather, it is a factor that maintains homeostasis during immune reactions. Prolactin is involved in stimulating the immune response, providing specific interference in inducing B-lymphocyte tolerance, enhancing the proliferative response to antigens and mitogens and increasing immunoglobulin and cytokine production, including of IL-1*β* [48–50]. This positive acute-phase protein induces IL-6 production through, e.g., peritoneal mesothelial cells, which additionally contributes to the local inflammation in endometriosis patients [3]. Through the action of IL-1*β* in promoting endometrial cells' angiogenesis and proliferation, it may play a key role in the development of endometriosis [51, 52]. Uric acid also indirectly contributes to inducing IL-1*β* synthesis [53]. Additionally, attention should be paid to UA dualism. Under physiological conditions, UA reflects the body's metabolic state and has antioxidant properties. It is responsible for approximately 60% of total antioxidant capacity and, along with other low-molecular-weight antioxidants such as total bilirubin, is the first line of antioxidant defense. However, given reduced availability of other antioxidants, it begins to act as an oxidative factor in various pathological processes. UA's role and the mechanism of its action in reproductive system disorders have not yet been elucidated [53, 54], although our studies have also emphasized its importance in advanced endometriosis. Moreover, both prolactin and uric acid in the blood serum can serve as biomarkers for the activity of some autoimmune diseases [49].

**Figure 5:**
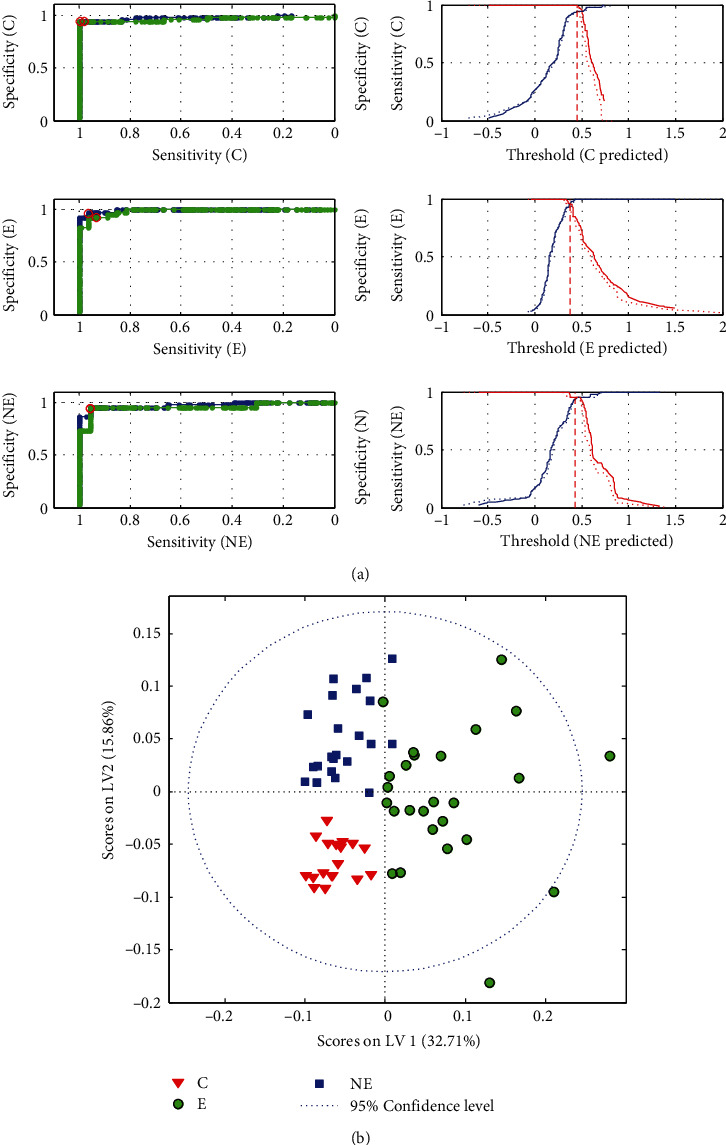
ROC curves (a) and scores plot (b) for the PLS-DA model based on the fused data. E: endometriosis; NE: nonendometriosis; C: control group of healthy women.

The constructed classifier had accuracy of 99-100%, while its sensitivity and specificity were 96-100% and 98-100%, respectively (Table 2). Notably, this model required only two LVs to reach the best performance, versus 4-5 PLS factors needed by PLS-DA models built separately for biochemical parameters or spectra. The obtained PLS-DA score plots and ROC curves are shown in Figure 5, while classification errors are presented in Fig. S14 in the Supplementary Materials.

The parameters of the classifiers obtained based on different data blocks occurred to be quite similar, as were their separation of the three groups of women. However, incorporating spectral data significantly improved the robustness of the elaborated models, in comparison with the values obtained for the models based on biochemical parameters only (Table 2). Since no specific marker of endometriosis has been found that would allow unequivocal diagnosis of the disease, combining spectral data with routinely determined biochemical parameters used to assess the state of the body could provide a tool for detecting women with a high probability of advanced endometriosis.

## 4. Conclusions

PLS-DA models were developed based on selected biochemical parameters and regions of FTIR ATR spectra of serum that could identify women at risk of advanced endometriosis, women with a developing inflammatory process with another origin, and healthy women. The sensitivity, specificity, and accuracy of the obtained models were 81-100%, 89-100%, and 91-100%, respectively. This study shows that infrared spectroscopy and discrimination analysis can be used to differentiate serum samples originating from women with advanced endometriosis and without endometriosis. Standardization of this method, based on the results obtained for a larger group of participants, likely will allow for effective endometriosis screening and diagnostics of this disease with advanced stages. One remaining challenge is still the development of classifiers able to detect the early stages of endometriosis.

## Figures and Tables

**Table 1:** Serum biochemical parameters for the studied groups.

	Endometriosis *n* = 29	Nonendometriosis *n* = 24	Control *n* = 18
	Mean ± SD	Mean ± SD	Mean ± SD
PRL (ng/mL)	29.01 ± 18.71	27.02 ± 18.62	12.84 ± 4.86
CA 125 (U/mL)	113.41 ± 129.42	23.64 ± 17.39	14.28 ± 7.40
IgG (mg/dL)	1065.46 ± 286.73	1078.22 ± 298.25	1237.28 ± 215.91
hsCRP (mg/L)	14.29 ± 19.45	13.88 ± 19.96	0.96 ± 1.19
Albumin (g/dL)	4.36 ± 0.64	4.13 ± 0.32	4.26 ± 0.19
Calcium (mg/dL)	9.62 ± 1.15	9.35 ± 0.49	9.29 ± 0.25
Magnesium (mg/dL)	2.44 ± 0.37	2.47 ± 0.18	2.22 ± 0.14
hsIL-1*β* (pg/ml)	0.57 ± 0.39	0.56 ± 0.46	0.27 ± 0.26
IL-6 (pg/ml)	19.33 ± 43.69	18.05 ± 34.09	1.47 ± 1.48
FRAP (mmol/L)	1.11 ± 0.26	1.18 ± 0.30	0.95 ± 0.22
AOPP (*μ*mol/L)	235.16 ± 150.41	181.78 ± 156.00	105.16 ± 49.24
YKL-40 (ng/mL)	685.22 ± 1246.58	403.29 ± 934.76	104.12 ± 154.07

AOPP: advanced protein oxidation products; CA 125: carcinoma antigen 125; FRAP: ferric-reducing antioxidant power; hsCRP: high sensitive C-reactive protein; hsIL-1*β*: high sensitive interleukin 1*β*; IgG: immunoglobulin G; IL-6: interleukin 6; PRL: prolactin; YKL-40: chitinase-3-like protein 1. Data presented in the table are a part of previously published study results [2, 26].

**Table 2:** Parameters of PLS-DA models.

Parameter	Biochemistry			FTIR ATR			Fused data		
	E	NE	C	E	NE	C	E	NE	C
Accuracy	92.2 (87.5)	92.2 (85.9)	90.6 (89.1)	98.6 (92.9)	97.1 (92.9)	98.6 (94.3)	98.5 (94.1)	98.5 (95.6)	100 (98.5)
Sensitivity (TPR)	80.8 (76.9)	90.0 (75.0)	94.4 (94.4)	96.6 (89.7)	95.8 (91.7)	100 (88.2)	96.3 (92.6)	100 (91.3)	100 (100)
Specificity (TNR)	100 (94.7)	93.2 (90.9)	89.1 (87.0)	100 (95.1)	97.8 (93.5)	98.1 (96.2)	100 (95.1)	97.8 (97.8)	100 (98.0)
Precision (PPV)	100 (90.9)	85.7 (78.9)	77.3 (73.9)	100 (92.9)	95.8 (88.0)	94.4 (88.2)	100 (92.6)	95.8 (95.5)	100 (94.7)
F1-score	89.4 (83.3)	87.8 (76.9)	85.0 (82.9)	98.2 (91.2)	95.8 (89.8)	97.1 (88.2)	98.1 (92.6)	97.9 (93.3)	100 (97.3)
Overall accuracy	87.5 (81.3)			97.3 (89.9)			98.5 (94.3)		

E: endometriosis; NE: nonendometriosis; C: control group of healthy women; TPR: true positive rate; TNR: true negative rate; PPV: positive predictive value. In parenthesis, the results of cross-validation are shown.

## Data Availability

The corresponding authors can provide the datasets for this study upon reasonable request.

## Abbreviations

ALB: Albumin
AOPP: Advanced protein oxidation products
BMI: Body mass index
C: Control group of healthy women
Ca: Calcium
CA 125: Carcinoma antigen 125
DA: Discriminant analysis
E: Endometriosis
E2: Estradiol
Fe: Iron
FRAP: Ferric-reducing antioxidant power
FTIR ATR: Fourier transform infrared spectroscopy-attenuated total reflectance
GLSW: General least squares weighting
GLU: Glucose
HDL: High-density lipoprotein cholesterol
hsCRP: High sensitive C-reactive protein
hsIL-1*β*: High sensitive interleukin 1*β*
IgG: Immunoglobulin G
IL-6: Interleukin 6
iPLS: Interval partial least squares
IR: Infrared spectroscopy
LDL: Low-density lipoprotein cholesterol
LV: Latent variable
Mg: Magnesium
NE: Nonendometriosis
PC: Principal component
PCA: Principal component analysis
PLS-DA: Partial least squares discriminant analysis
PPV: Positive predictive value
PRL: Prolactin
rAFS: Revised American Fertility Society classification
ROC: Receiver operating characteristic curve
SD: Standard deviation
SIRT3: Sirtuin 3
SIRT5: Sirtuin 5
SIRT6: Sirtuin 6
TAS: Total antioxidant status
TE: Telomerase
TG: Triglycerides
TNR: True negative rate
T-P: Total protein
TPR: True positive rate
T-BIL: Total bilirubin
T-CHOL: Total cholesterol
UA: Uric acid
VIP: Variable importance in projection
YKL-40: Chitinase-3-like protein 1.

## Supplementary Materials

Supplementary Materials Supplementary Materials Table S1: serum parameters. Table S2: confusion matrix for PLS-DA models after iPLS variable selection. Fig. S1: PCA scores based on biochemical parameters of serum samples. Fig. S2: PCA of biochemical serum parameters obtained with GLSW pretreatment. Fig. S3: VIP scores for PLS-DA modeling of biochemical data of serum samples. Fig. S4: PLS-DA of serum biochemical parameters. Fig. S5: Average FTIR ATR spectra of serum samples for three studied groups and second derivatives of spectra. Fig. S6: ATR difference spectra of average serum samples with ±SD of absorbance. Fig. S7: IR spectra of serum sample dried on an ATR crystal collected in 10 min intervals. Fig. S8: PCA for ATR spectra of serum sample during drying and thin film formation. Fig. S9: PCA scores for ATR spectra of serum samples obtained based on 700-1450 cm^−1^ spectral range. Fig. S10: PLS-DA scores for ATR spectra of serum samples obtained based on 700-1450 cm^−1^ spectral range. Fig. S11: VIP scores of PLS-DA and iPLS variable selection for ATR data in the 700-1450 cm^−1^ spectral range. Fig. S12: PLS-DA of ATR spectra of sera-ROC plots for all three groups of samples and calibration/cross-validation errors for the model constructed applying variables selected by iPLS. Fig. S13: ATR inputs selected by PCA for fused data modeling. Fig. S14: errors of classification for PLS-DA model constructed on the basis of fused data.
