# Supplementary material for: ATR-IR Spectroscopy Application to Diagnostic Screening of Advanced Endometriosis
Source: Oxid Med Cell Longev. 2022 Jun 6;2022:4777434. doi: 10.1155/2022/4777434 (PMC9192200; doi:10.1155/2022/4777434)
Supplement: Supplementary Materials — Table S1: serum parameters. Table S2: confusion matrix for PLS-DA models after iPLS variable selection. Fig. S1: PCA scores based on biochemical parameters of serum samples. Fig. S2: PCA of biochemical serum parameters obtained with GLSW pretreatment. Fig. S3: VIP scores for PLS-DA modeling of biochemical data of serum samples. Fig. S4: PLS-DA of serum biochemical parameters. Fig. S5: Average FTIR ATR spectra of serum samples for three studied groups and second derivatives of spectra. Fig. S6: ATR difference spectra of average serum samples with ±SD of absorbance. Fig. S7: IR spectra of serum sample dried on an ATR crystal collected in 10 min intervals. Fig. S8: PCA for ATR spectra of serum sample during drying and thin film formation. Fig. S9: PCA scores for ATR spectra of serum samples obtained based on 700-1450 cm−1 spectral range. Fig. S10: PLS-DA scores for ATR spectra of serum samples obtained based on 700-1450 cm−1 spectral range. Fig. S11: VIP scores of PLS-DA and iPLS variable selection for ATR data in the 700-1450 cm−1 spectral range. Fig. S12: PLS-DA of ATR spectra of sera-ROC plots for all three groups of samples and calibration/cross-validation errors for the model constructed applying variables selected by iPLS. Fig. S13: ATR inputs selected by PCA for fused data modeling. Fig. S14: errors of classification for PLS-DA model constructed on the basis of fused data. [file 4777434.f1.pdf]

ATR-IR spectroscopy application to diagnostic screening of advanced endometriosis

Izabela Kokot[1*], Sylwester Mazurek[2*], Agnieszka Piwowar[3], Roman Szostak[2], Marcin Jędryka[4,5], Ewa Maria Kratz[1]

[1] Department of Laboratory Diagnostics, Division of Laboratory Diagnostics, Faculty of Pharmacy, Wroclaw Medical University, Borowska Street 211A, 50-556 Wroclaw, Poland

[2] Faculty of Chemistry, University of Wrocław, F. Joliot-Curie 14, 50-383 Wrocław, Poland

[3] Department of Toxicology, Faculty of Pharmacy, Wroclaw Medical University, Borowska Street 211, 50-556 Wroclaw, Poland

[4] Department of Oncology, Gynecological Oncology Clinic, Faculty of Medicine, Hirszfeld Square 12, 53-413 Wroclaw Medical University, Wroclaw, Poland

[5] Department of Oncological Gynecology, Wroclaw Comprehensive Cancer Center, Hirszfeld Square 12, 53-413 Wroclaw, Poland

Correspondence to: Izabela Kokot: izabela.kokot@umed.wroc.pl or Sylwester Mazurek: sylwester.mazurek@chem.uni.wroc.pl

Supporting Informations

List of content:

Table S1. Serum parameters
Table S2. Confusion matrix for PLS-DA models after iPLS variable selection
Fig. S1. PCA scores based on biochemical parameters of serum samples
Fig. S2. PCA of biochemical serum parameters obtained with GLSW pre-treatment
Fig. S3. VIP scores for PLS-DA modeling of biochemical data of serum samples
Fig. S4. PLS-DA of serum biochemical parameters
Fig. S5. Average FTIR ATR spectra of serum samples for three studied groups and second derivatives of spectra
Fig. S6. ATR difference spectra of average serum samples with +/-SD of absorbance
Fig. S7. IR spectra of serum sample dried on an ATR crystal collected in 10-min. intervals
Fig. S8. PCA for ATR spectra of serum sample during drying and thin film formation
Fig. S9. PCA scores for ATR spectra of serum samples obtained based on 700-1450 cm^{-1} spectral range
Fig. S10. PLS-DA scores for ATR spectra of serum samples obtained based on 700-1450 cm^{-1} spectral range
Fig. S11. VIP scores of PLS-DA and iPLS variable selection for ATR data in the 700-1450 cm^{-1} spectral range
Fig. S12. PLS-DA of ATR spectra of sera - ROC plots for all three groups of samples and calibration/cross-validation errors for the model constructed applying variables selected by iPLS
Fig. S13. ATR inputs selected by PCA for fused data modeling
Fig. S14. Errors of classification for PLS-DA model constructed on the basis of fused data

Table S1. Serum parameters

	Endometriosis n = 29	Non-endometriosis n = 24	Control n = 18
	**Mean ± SD**	**Mean ± SD**	**Mean ± SD**
Estradiol [pg/mL]	83.63 ± 83.93	96.59 ± 81.74	77.52 ± 65.17
PRL [ng/mL]	29.01 ± 18.71	27.02 ± 18.62	12.84 ± 4.86
CA 125 [U/mL]	113.41 ± 129.42	23.64 ± 17.39	14.28 ± 7.40
IgG [mg/dL]	1065.46 ± 286.73	1078.22 ± 298.25	1237.28 ± 215.91
TAS [mmol/L]	1.50 ± 0.18	1.61 ± 0.27	1.60 ± 0.22
hsCRP [mg/L]	14.29 ± 19.45	13.88 ± 19.96	0.96 ± 1.19
Glucose [mg/dL]	77.79 ± 25.97	69.79 ± 17.91	86.24 ± 10.63
Total protein [g/dL]	6.89 ± 1.32	6.61 ± 0.68	6.77 ± 0.38
Albumin [g/dL]	4.36 ± 0.64	4.13 ± 0.32	4.26 ± 0.19
T-BIL [mg/dL]	0.54 ± 0.37	0.65 ± 0.45	0.59 ± 0.24
Calcium [mg/dL]	9.62 ± 1.15	9.35 ± 0.49	9.29 ± 0.25
Magnesium [mg/dL]	2.44 ± 0.37	2.47 ± 0.18	2.22 ± 0.14
Iron [µg/dL]	104.10 ± 60.76	107.21 ± 74.27	105.89 ± 38.24
T-CHOL [mg/dL]	197.83 ± 48.31	181.50 ± 28.66	190.67 ± 36.63
HDL [mg/dL]	53.79 ± 12.84	53.96 ± 10.38	55.44 ± 12.52
TG [mg/dL]	105.62 ± 44.41	111.79 ± 78.38	102.06 ± 42.21
Uric Acid [mg/dL]	4.78 ± 1.02	4.85 ± 1.38	4.72 ± 0.95
LDL [mg/dL]	122.86 ± 37.35	105.08 ± 23.54	114.72 ± 30.54
hsIL-1β [pg/ml]	0.57 ± 0.39	0.56 ± 0.46	0.27 ± 0.26
IL-6 [pg/ml]	19.33 ± 43.69	18.05 ± 34.09	1.47 ± 1.48
FRAP [mmol/L]	1.11 ± 0.26	1.18 ± 0.30	0.95 ± 0.22
AOPP [µmol/L]	235.16 ± 150.41	181.78 ± 156.00	105.16 ± 49.24
YKL-40 [ng/ml]	685.22 ± 1246.58	403.29 ± 934.76	104.12 ± 154.07
SIRT3 [ng/mL]	12.32 ± 7.59	11.11 ± 6.80	11.22 ± 6.03
SIRT5 [ng/mL]	8.21 ± 4.41	9.61 ± 5.72	8.49 ± 6.04
SIRT6 [ng/mL]	12.32 ± 16.84	15.88 ± 18.74	8.45 ± 10.95
Telomerase [ng/mL]	1.61 ± 1.88	1.82 ± 2.96	0.39 ± 0.52

AOPP — advanced protein oxidation products; CA 125 — carcinoma antigen 125; FRAP — ferric reducing antioxidant power; HDL — high-density lipoprotein cholesterol; hsCRP — high sensitive C-reactive protein; hsIL-1β — high sensitive interleukin 1β; IgG — immunoglobulin G; IL-6 — interleukin 6; LDL — low-density lipoprotein cholesterol; PRL — prolactin; SIRT3 — sirtuin 3; SIRT5 — sirtuin 5; SIRT6 — sirtuin 6; TAS — total antioxidant status; TG — triglycerides; T-BIL — total bilirubin; T-CHOL — total cholesterol; YKL-40 — chitinase-3-like protein 1.

Table S2. Confusion matrix for PLS-DA models after iPLS variable selection

Data set		Calibration			Cross-validation		
		Actual C	Actual E	Actual NE	Actual C	Actual E	Actual NE
Biochemical parameters	Predicted as C	17	3	2	17	3	3
	Predicted as E	0	21	0	0	20	2
	Predicted as NE	1	2	18	1	3	15
ATR spectra	Predicted as C	17	0	1	15	1	1
	Predicted as E	0	28	0	1	26	1
	Predicted as NE	0	1	23	1	2	22
Fused data	Predicted as C	18	1	1	18	2	1
	Predicted as E	0	25	1	0	24	3
	Predicted as NE	0	3	22	0	3	20

E – endometriosis, NE – non-endometriosis, C – control group of healthy women.

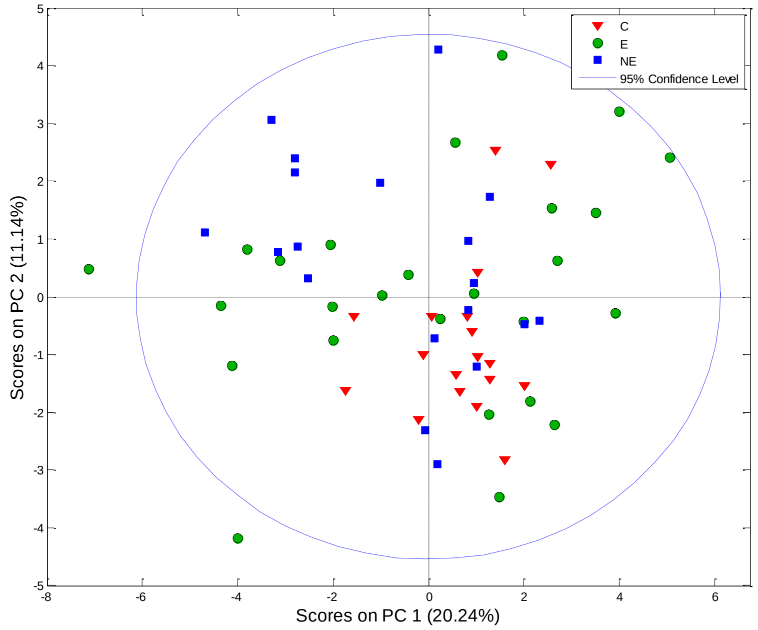

Figure S1. PCA scores based on biochemical parameters of serum samples. E – endometriosis, NE – non-endometriosis, C – control group of healthy women.

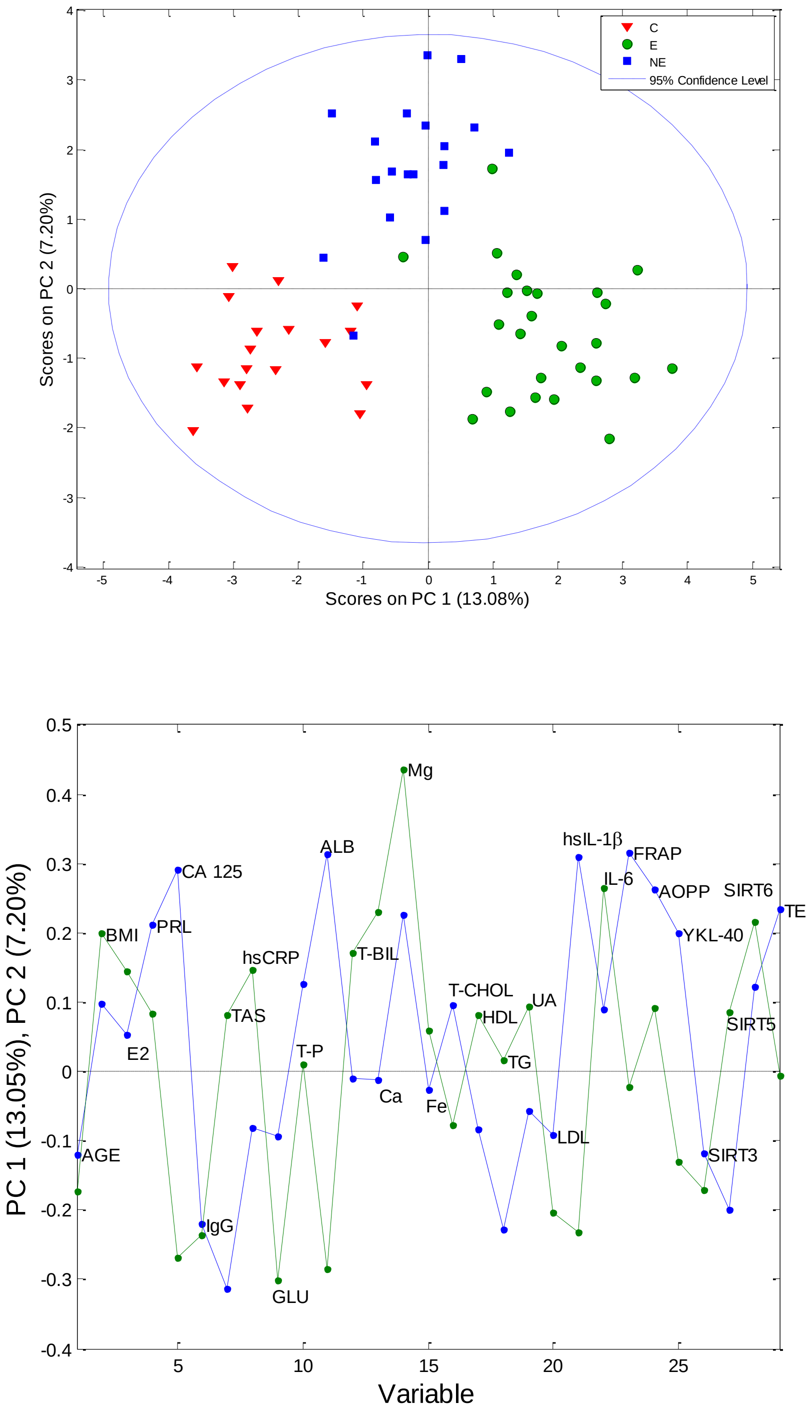

Figure S2. PCA of biochemical serum parameters obtained with GLSW pre-treatment; scores (top) and loadings (bottom). E – endometriosis, NE – non-endometriosis, C – control group of healthy women. ALB — albumin; AOPP — advanced protein oxidation products; BMI — body mass index; Ca — calcium; CA 125 — carcinoma antigen 125; E2 – estradiol; Fe — iron; FRAP — ferric reducing antioxidant power; GLSW — general least squares weighting; GLU — glucose; HDL — high-density lipoprotein cholesterol; hsCRP — high sensitive C-reactive protein; hsIL-1β — high sensitive interleukin 1β; IgG — immunoglobulin G; IL-6 — interleukin 6; LDL — low-density lipoprotein cholesterol; Mg — magnesium; PRL — prolactin; SIRT3 — sirtuin 3; SIRT5 — sirtuin 5; SIRT6 — sirtuin 6; TAS — total antioxidant status; TE — telomerase; TG — triglycerides; T-P — total protein; T-BIL — total bilirubin; T-CHOL — total cholesterol; UA — uric acid; YKL-40 — chitinase-3-like protein 1.

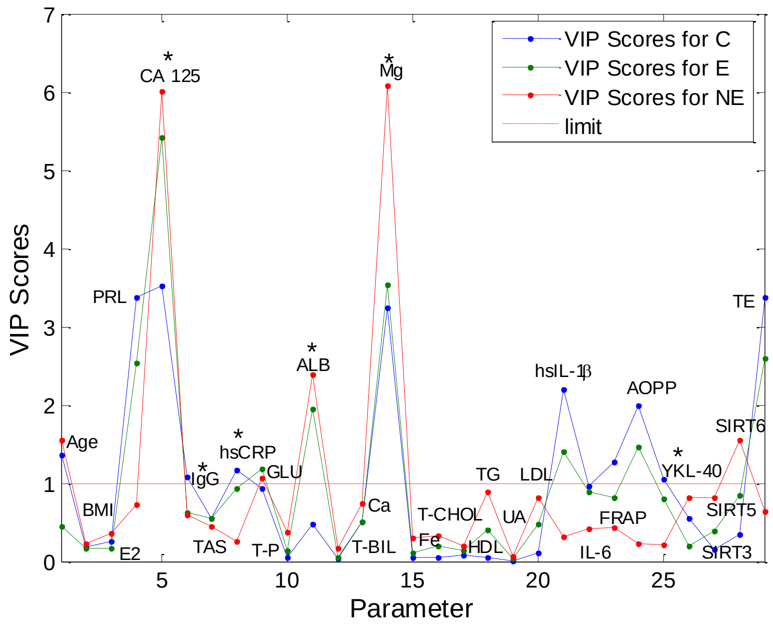

Figure S3. VIP scores for PLS-DA modeling of biochemical data of serum samples; * indicates variables selected by the iPLS. E – endometriosis, NE – non-endometriosis, C – control group of healthy women. ALB — albumin; AOPP — advanced protein oxidation products; BMI — body mass index; Ca — calcium; CA 125 — carcinoma antigen 125; E2 – estradiol; Fe — iron; FRAP — ferric reducing antioxidant power; GLU — glucose; HDL — high-density lipoprotein cholesterol; hsCRP — high sensitive C-reactive protein; hsIL-1β — high sensitive interleukin 1β; IgG — immunoglobulin G; IL-6 — interleukin 6; LDL — low-density lipoprotein cholesterol; Mg — magnesium; PRL — prolactin; SIRT3 — sirtuin 3; SIRT5 — sirtuin 5; SIRT6 — sirtuin 6; TAS — total antioxidant status; TE — telomerase; TG — triglycerides; T-P — total protein; T-BIL — total bilirubin; T-CHOL — total cholesterol; UA — uric acid; YKL-40 — Chitinase-3-like protein 1.

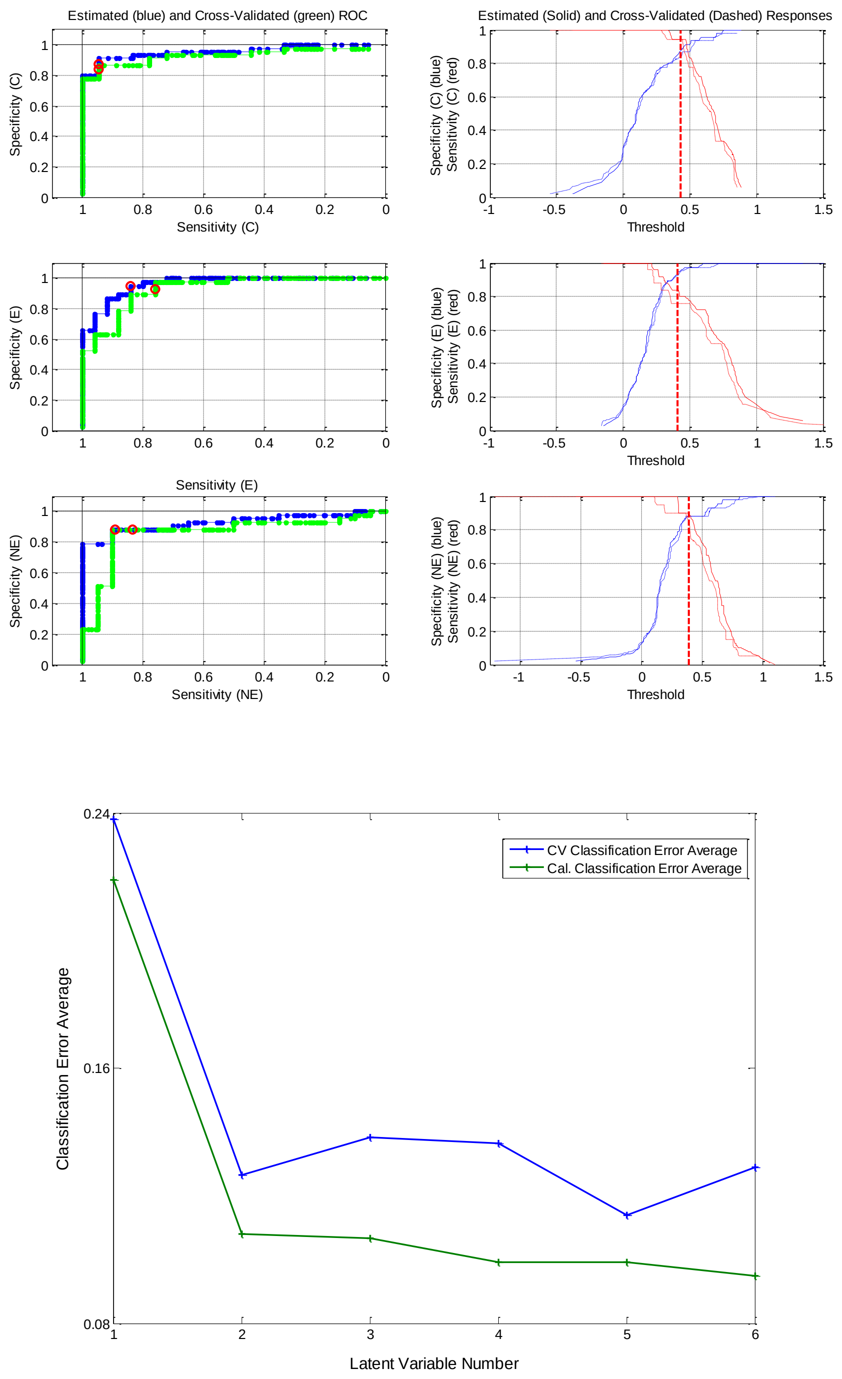

Figure S4. PLS-DA of serum biochemical parameters; ROC plots for three groups of samples (top); calibration and cross-validation errors for the model constructed applying variables selected by iPLS (bottom). E – endometriosis, NE – non-endometriosis, C – control group of healthy women.

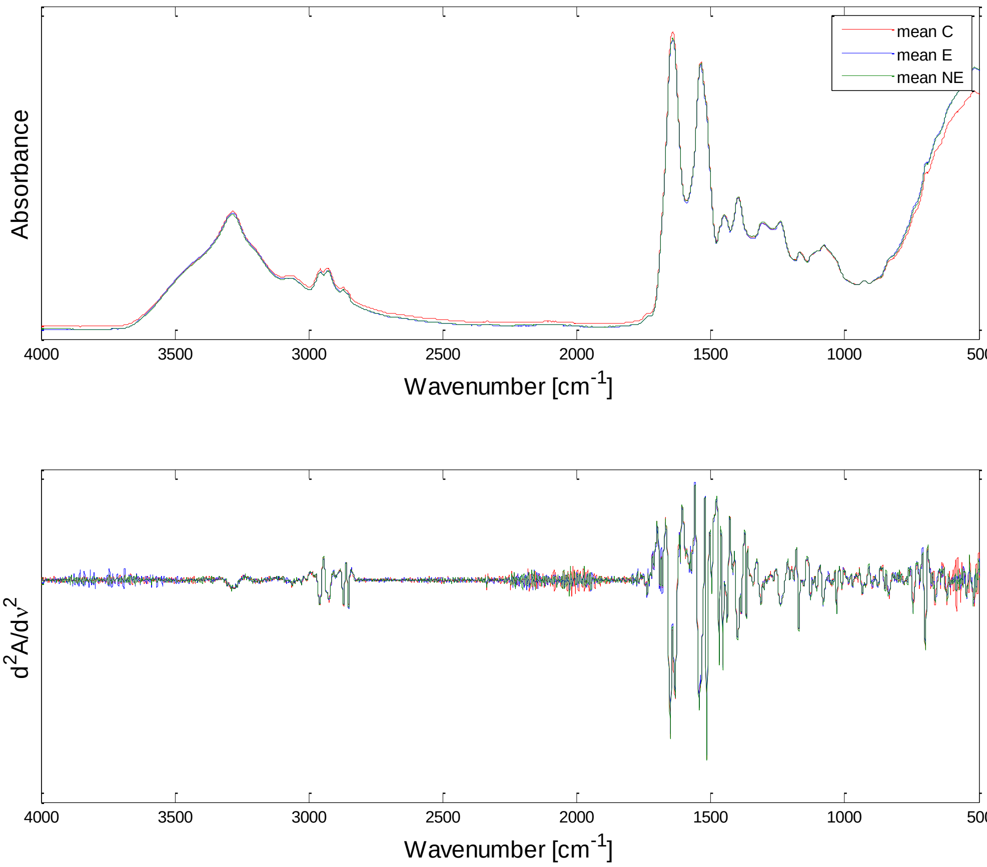

Figure S5. Average FTIR ATR spectra of serum samples for three studied groups (top) and second derivatives of spectra (bottom). E – endometriosis, NE – non-endometriosis, C – control group of healthy women.

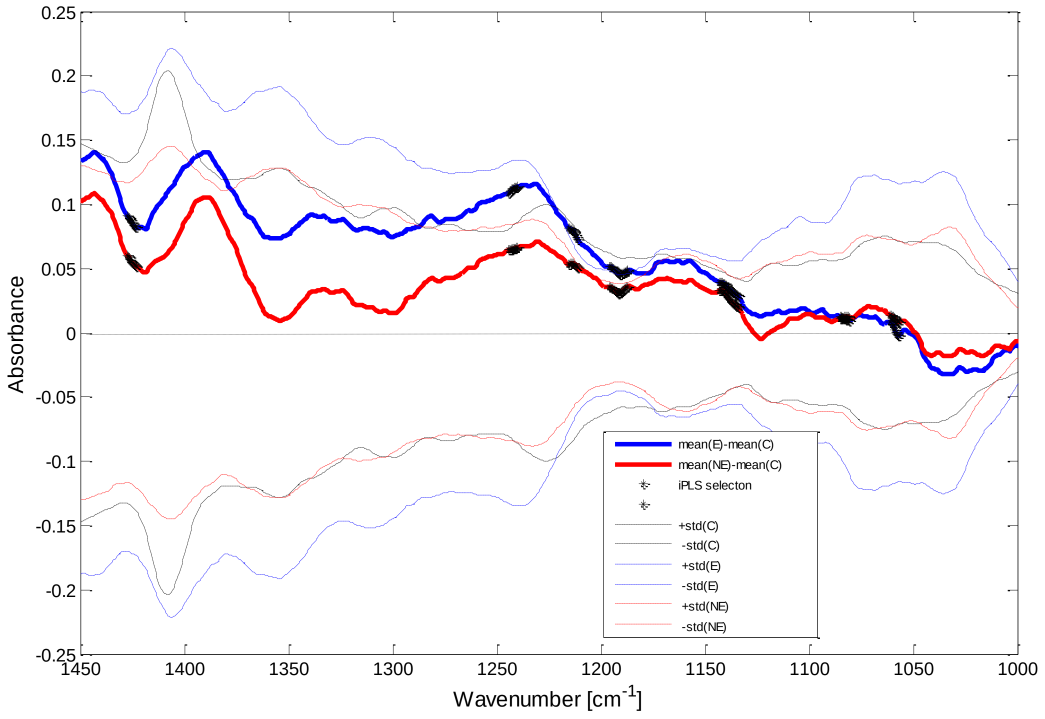

Figure S6. Difference spectra between the mean of sera samples with +/- standard deviation of signals' intensities. E – endometriosis, NE – non-endometriosis, C – control group of healthy women.

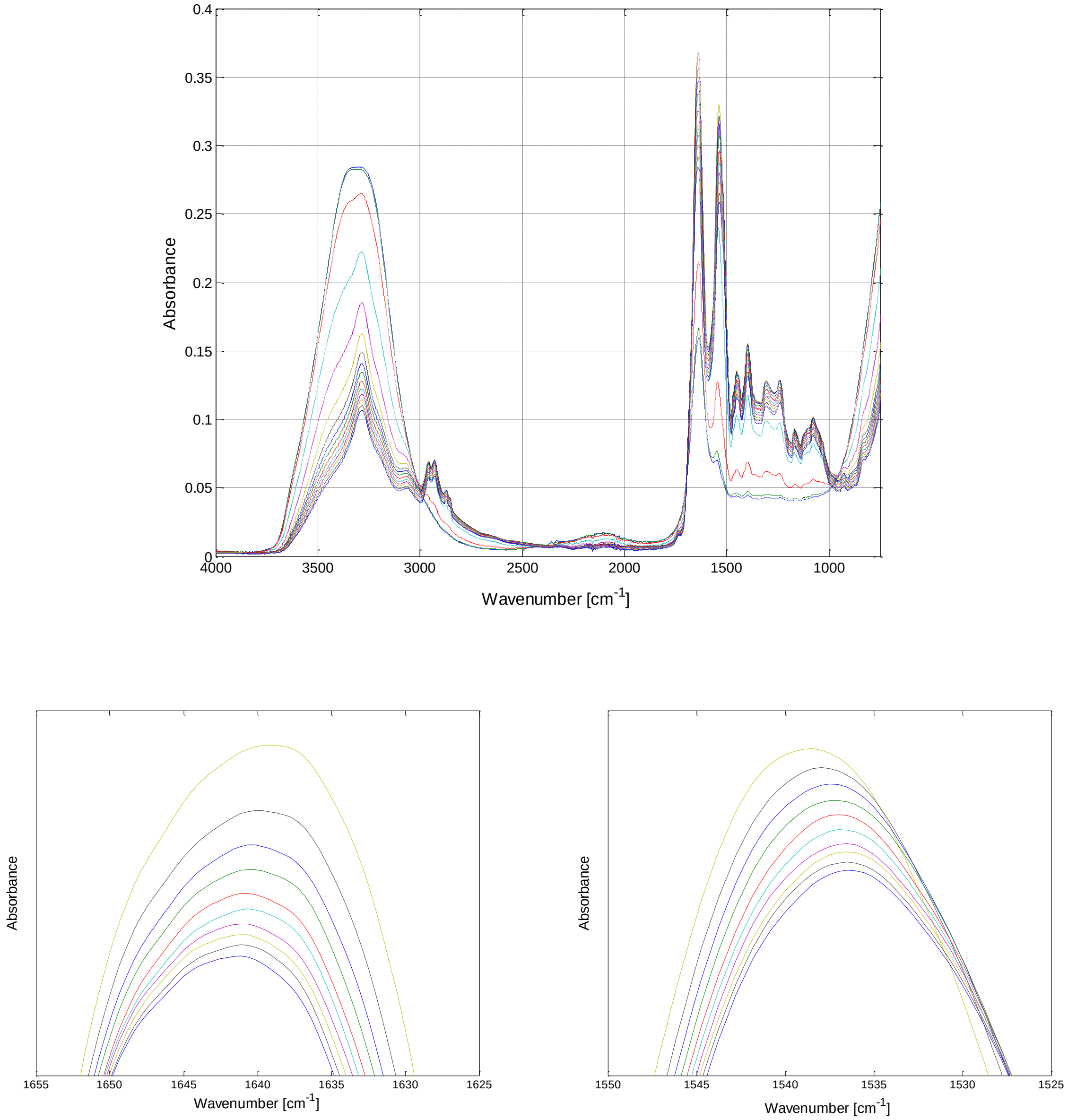

Figure S7. IR spectra of serum sample dried on an ATR crystal collected in 10-min. intervals (top); change of the peak position of the Amide I (left) and Amide II (right) bands during thin film formation (bottom). E – endometriosis, NE – non-endometriosis, C – control group of healthy women.

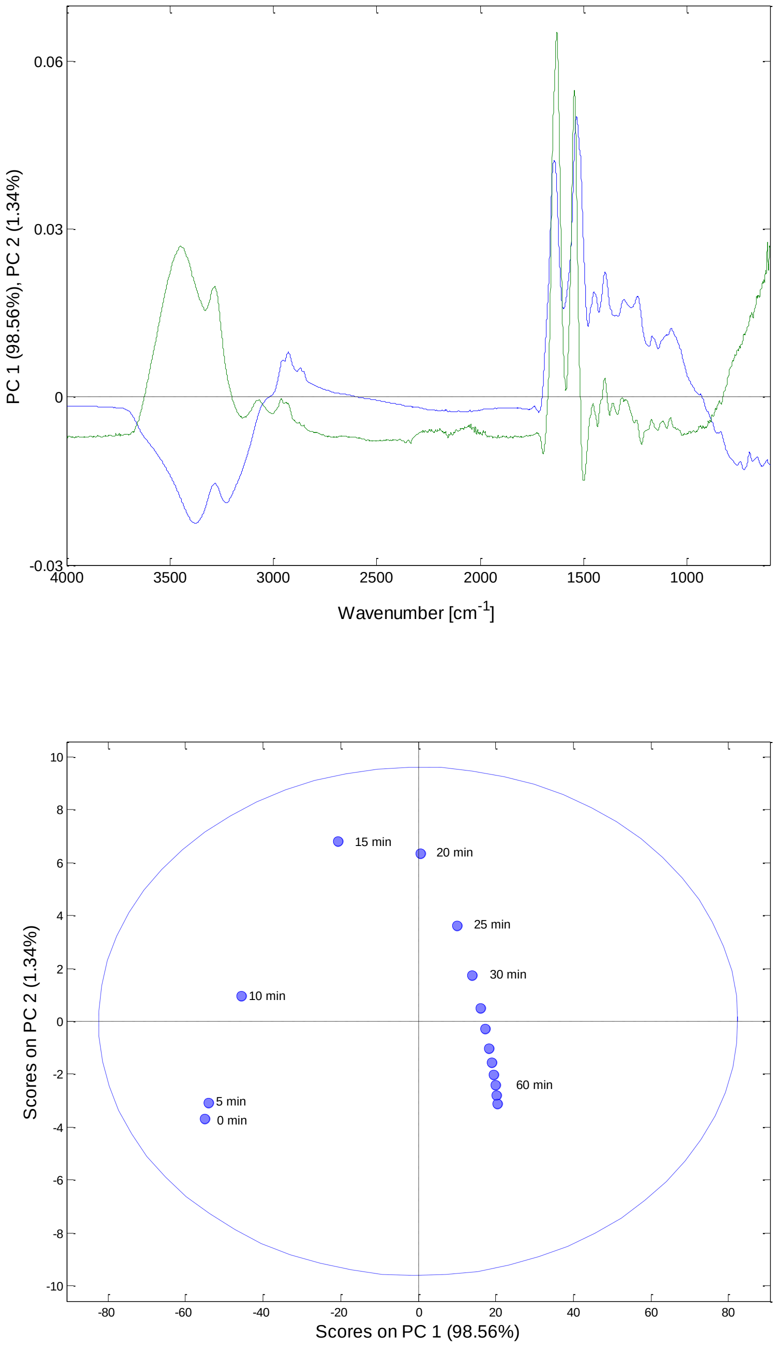

Figure S8. PCA for ATR spectra of serum sample during drying and thin film formation: loadings of two first principal components (top) and scores plots (bottom).

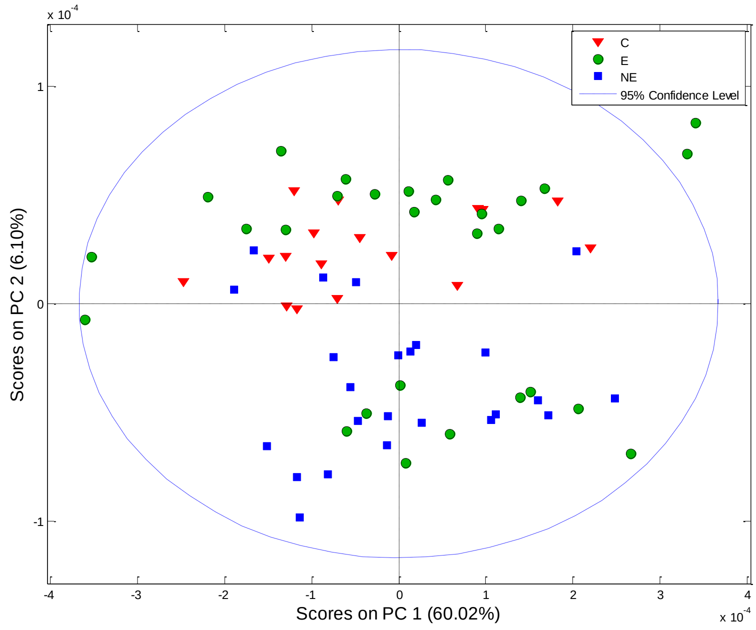

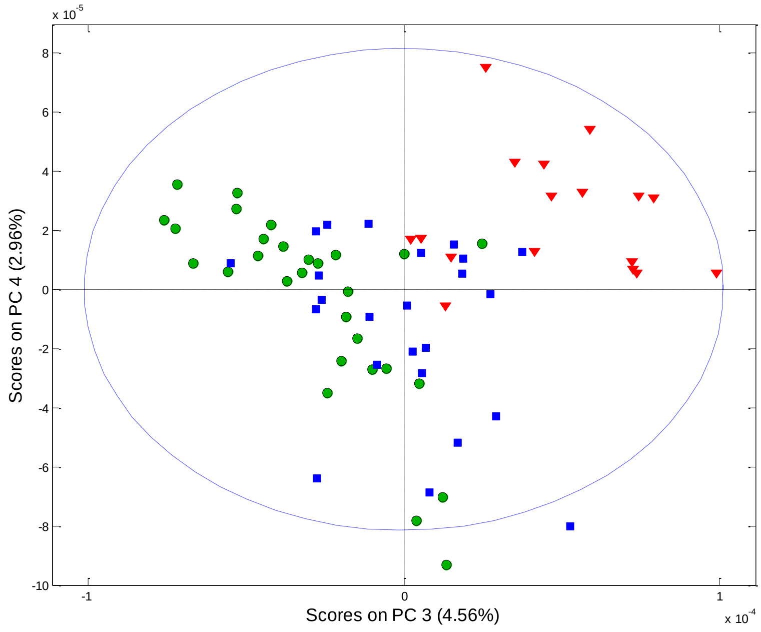

Figure S9. PCA scores for ATR spectra of serum samples obtained based on 700-1450 cm^{-1} spectral range; loadings of two first principal components (top) and third and fourth principal components (bottom). E – endometriosis, NE – non-endometriosis, C – control group of healthy women.

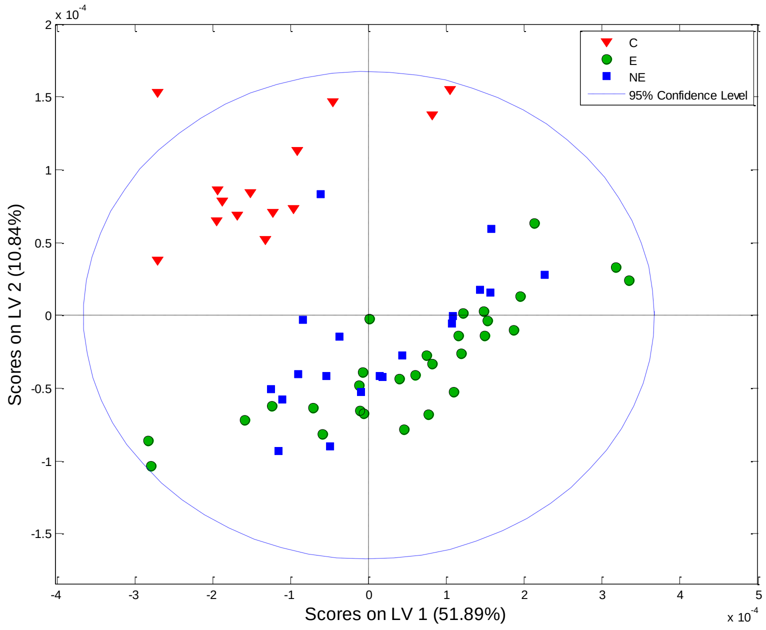

Figure S10. PLS-DA scores for ATR spectra of serum samples obtained based on 700-1450 cm^{-1} spectral range. E – endometriosis, NE – non-endometriosis, C – control group of healthy women.

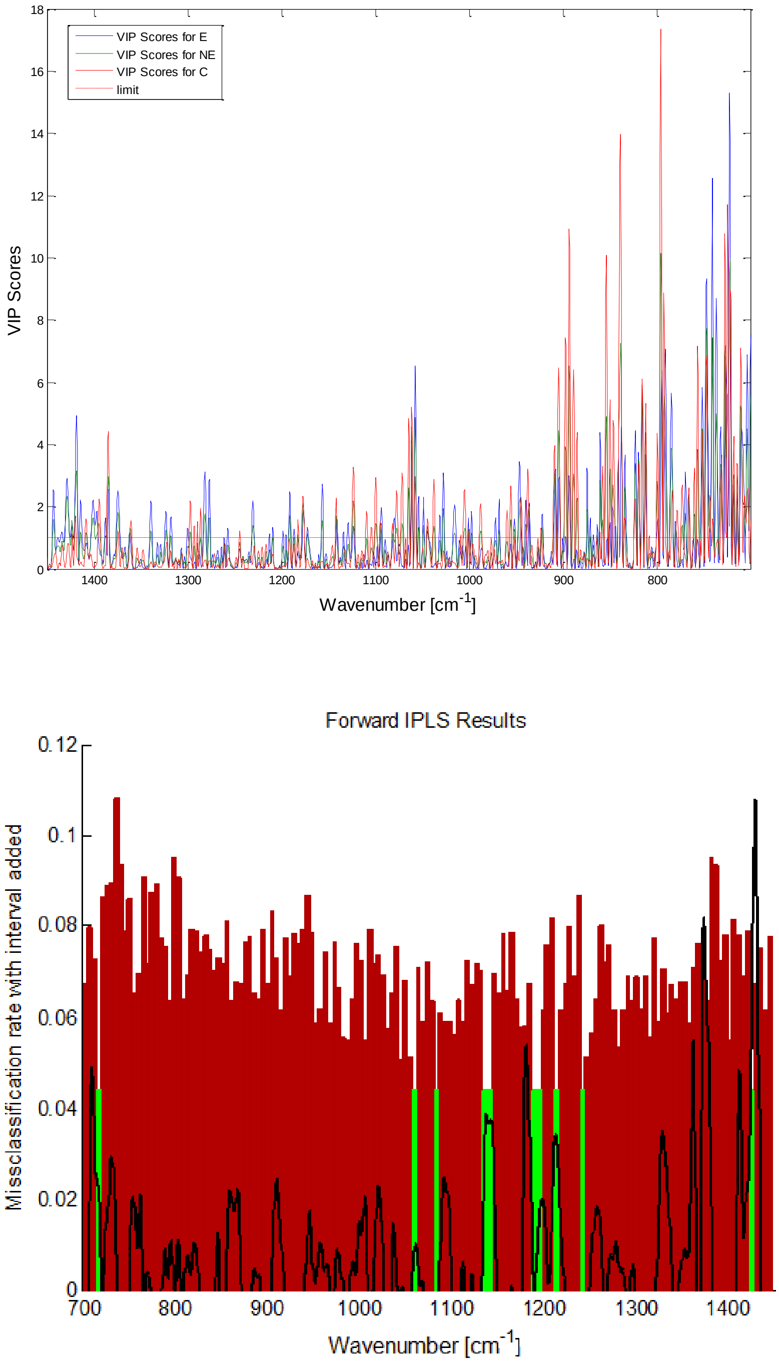

Figure S11. VIP scores of PLS-DA (top) and iPLS variable selection (bottom) for ATR data in the 700-1450 cm^{-1} spectral range. E – endometriosis, NE – non-endometriosis, C – control group of healthy women.

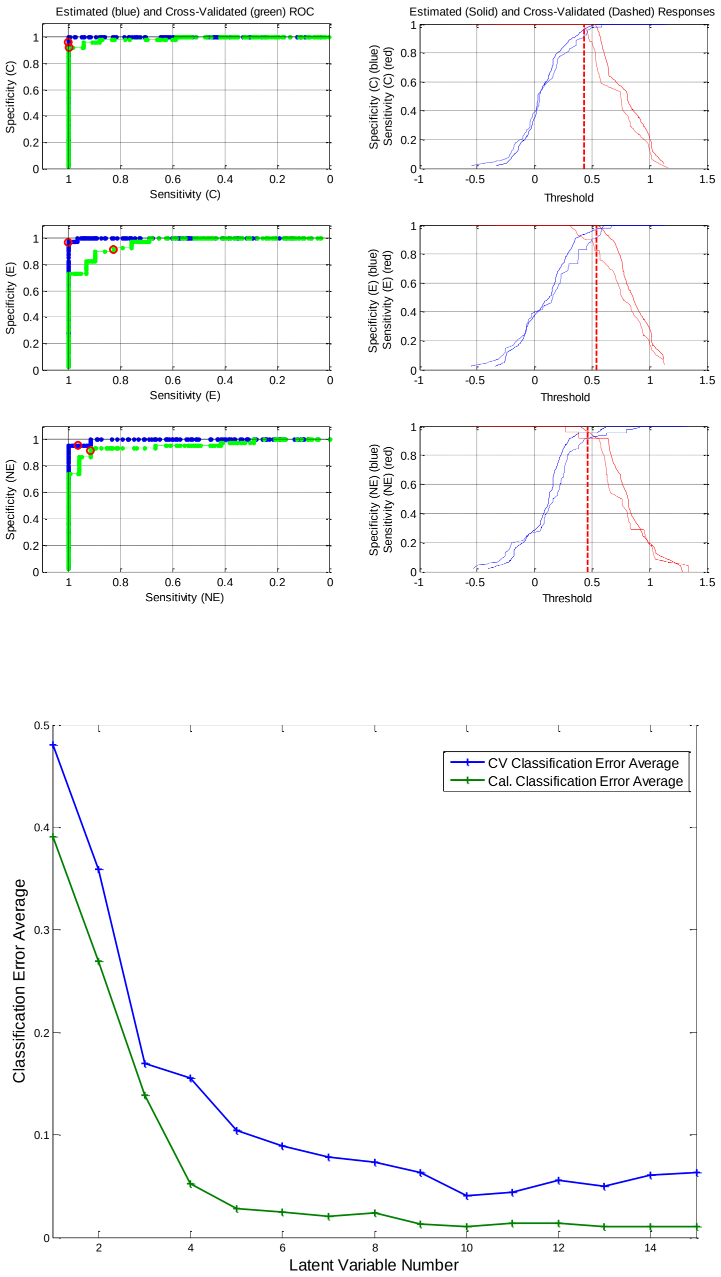

Figure S12. PLS-DA of ATR spectra of sera - ROC plots for all three groups of samples (top) and calibration/cross-validation errors for the model constructed applying variables selected by iPLS (bottom). E – endometriosis, NE – non-endometriosis, C – control group of healthy women.

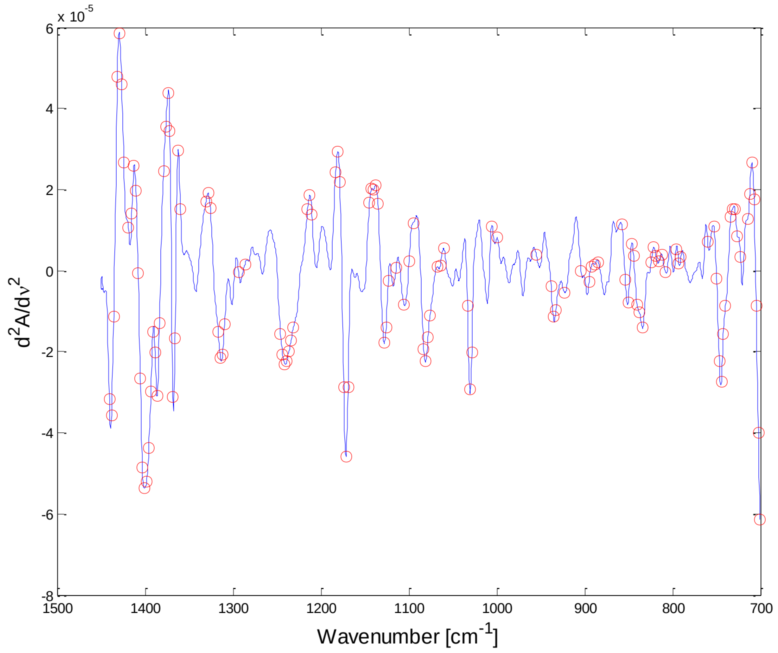

Figure S13. ATR inputs selected by PCA for fused data modeling.

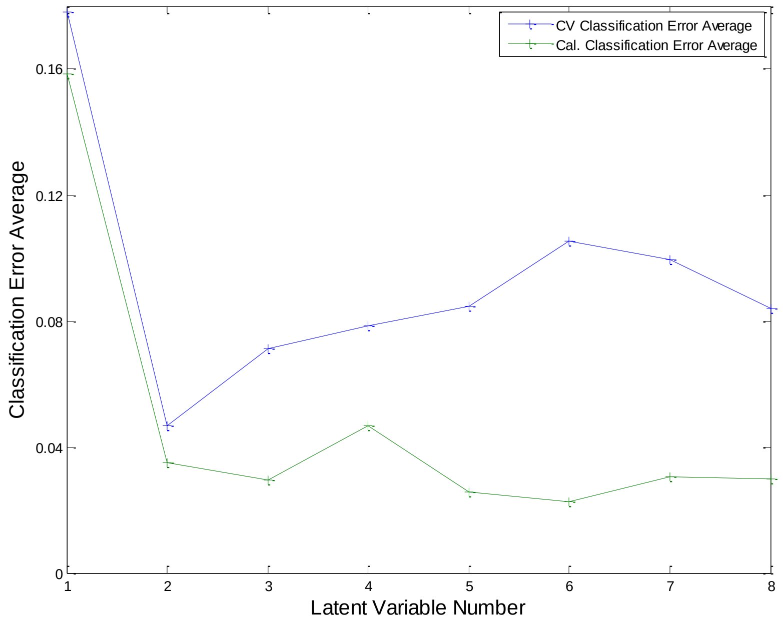

Figure S14. Errors of classification for PLS-DA model constructed on the basis of fused data.
